# Polydopamine Nanoparticles for Combined Chemo- and Photothermal Cancer Therapy

**DOI:** 10.3390/nano7070160

**Published:** 2017-06-29

**Authors:** Zhijun Zhu, Ming Su

**Affiliations:** 1 Department of Chemical Engineering, Northeastern University, Boston, MA 02115, USA; zhuzhujun001@gmail.com; 2 Wenzhou Institute of Biomaterials and Engineering, Wenzhou Medical University, Chinese Academy of Science, Wenzhou 325001, China

**Keywords:** polydopamine nanoparticles, photothermal, chemotherapy, cisplatin

## Abstract

Cancer therapy with two different modalities can enhance treatment efficacy and reduce side effects. This paper describes a new method for combined chemo- and photothermal therapy of cancer using poly dopamine nanoparticles (PDA-NPs), where PDA-NPs serve not only as a photothermal agent with strong near infrared absorbance and high energy conversion efficiency, but also as a carrier to deliver cisplatin via interaction between cisplatin and catechol groups on PDA-NPs. Polyethylene glycol (PEG) was introduced through Michael addition reaction to improve the stability of PDA-NPs in physiological condition. A remarkable synergistic therapeutic effect has been achieved compared with respective single treatments. This work suggests that the PDA-based nanoplatform can be a universal scaffold for combined chemo- and photothermal therapy of cancer.

## 1. Introduction

Cisplatin that contains platinum is used widely as a means of chemotherapy of cancer [1,2], but its clinical use is limited by its severe toxic effect due to indiscriminate accumulation in normal and cancerous tissues, nonspecific interactions with extra and intracellular proteins, and drug resistance [3]. In parallel to efforts of mitigating toxicity of cisplatin by modifying its chemical structure, an alternate approach is to use delivery vehicles that could overcome these limitations and specifically target cancerous cells [4]. Many carrier systems such as gold [5,6], carbon [7,8], silica [9,10], and polymer [11,12] have been developed, but the long-term toxicity of these carriers remains an issue [13]. A nature-inspired biopolymer, melanin-liked polydopamine (PDA) that has excellent biocompatibility [14,15,16], and free-radical-scavenging activity [17,18,19], has been explored as coating materials for gold nanorods [20,21], iron oxide nanoparticles [15,22,23,24], and as a substrate for photothermal agent [13], but the photothermal property of PDA nanoparticles has been overlooked. As a kind of semiconducting polymer [25], with a photothermal energy conversion efficiency of 40%, PDA-NPs have shown promising applications in photothermal-based cancer therapy, which is emerging as a powerful technique in cancer therapy due to localized treatment and minimal invasiveness [26,27,28,29].

This paper reports a PDA-NPs based therapeutic platform, where PDA-NPs loaded with anticancer drug cisplatin have been prepared through a mild method. PDA-NPs serve not only as a photothermal agent due to strong near infrared absorption and high photothermal energy conversion efficiency, but also as a carrier to load cisplatin via the interaction between cisplatin and catechol groups on PDA-NPs. Conjugation of cisplatin onto PDA-NPs has been achieved by mixing PDA-NPs and cisplatin in minutes. Polyethylene glycol (PEG), as a Food and Drug Administration (FDA) approved polymer has been introduced via a Michael addition reaction to improve the biocompatibility of the nanoplatform and prolong the circulation time of PDA-NPs in physiological condition. The anticancer drug cisplatin has been loaded onto PDA-NPs through the chelation interaction between cisplatin and catechol groups on PDA-NPs. A remarkable synergistic therapeutic effect is observed when compared with respective single treatments. This work suggests that PDA-NPs can serve as a drug delivery platform for combined chemo- and photothermal therapy.

## 2. Materials and Methods

Sodium dihydrogen phosphate (NaH_2_PO_4_), sodium hydrogen phosphate (Na_2_HPO_4_), ethanol and ammonia aqueous solution (28–32%) were from Deutsche Bahn (Berlin, Germany). Dulbecco’s modified eagle’s medium (DMEM), fetal bovine serum (FBS) and phosphate buffered saline (PBS) were from Corning. Methyl thiazolyl tetrazolium (MTT), cis-diamminedichlorido-platinum (II) (cisplatin, CP) and *o*-Phenylenediamine (OPDA) were obtained from Alfa (Tewksbury, MA, USA). Dimethylsulfoxide (DMSO), dopamine hydrochloride, poly(ethylene glycol) methyl ether thiol (PEG-SH, molecular weight 5000) and dialysis tube (MWCO = 2000 Da) were from Sigma (St. Louis, MO, USA).

PDA-NPs were made by injecting 2 mL of water containing 0.25 g of dopamine into a mixture of 20 mL of ethanol, 45 mL of water, and 1.5 mL of ammonia aqueous solution [26,30]. The mixture was stirred at room temperature for 24 h, followed by centrifugation with the addition of ethanol and washing with water. The PDA-NPs were modified with PEG by mixing 2 mL of PDA-NPs (4 mg) with 0.4 mL of mPEG-SH (8 mg) in 10 mM Tris buffer (pH 8.5) with overnight stirring. The PEGylated PDA-NPs were further purified by repeat centrifugation and dispersed in water. A JEOL JEM-1010 transmission electron microscope (TEM, JEOL, Tokyo, Japan) with 80 kV accelerating voltage was used to collect TEM images. The hydrated size and zeta potential were measured on a Malvern zetesizer (ZS6300, Malvern, Worcestershire, UK). UV-vis spectra were taken on Varian Cary 4000 spectrophotometer (Agilent, Santa Clara, CA, USA).

An amount of 20 mg of cisplatin was added into an aqueous solution containing 17 mg of silver nitrate (10 mg/mL). The mixture was stirred in the dark for 24 h at room temperature. After moving white precipitation (AgCl) by centrifugation at 10,000 rpm for 30 min, cis-diamminediaqua platinum (II) solution was obtained, stored in the dark at 4 °C and used up within 24 h. Cisplatin was loaded onto PDA-NPs by mixing 2 mL of PDA-NPs solution with cisplatin of desired amount under stirring for 30 min (denoted as PDA-PEG-CP), followed by centrifugation to remove extra drug.

The releasing profile of cisplatin was determined as follows. An amount of 5 mL of 2 mg/mL PDA-PEG-CP dispersion was loaded in a 2000 Da dialysis tube at 37 °C against 10 mL of PBS (10 mM) at different pH (6.0 or 7.4), respectively. After a certain time, 200 μL of dialysis solution was sampled, and 200 μL of fresh buffer was added. The total dialysis solutions were replaced with 10 mL fresh buffer at 6 h, 12 h, 24 h and 48 h, respectively. The amount of released cisplatin was measured via a modified literature procedure as follows [31]. Different aliquots (0.02 mL, 0.05 mL, 0.08 mL, 0.1 mL and 0.15 mL of 10 μg/mL, and 0.03 mL, 0.05 mL, 0.1 mL, 0.15 mL and 0.2 mL of 100 μg/mL) of cisplatin aqueous solution were diluted to 200 μL with water, followed by adding 200 μL of 0.7 mg/mL OPDA (freshly prepared in dimethylformamide (DMF)) and another 200 μL of phosphate buffer (0.1 M, pH 6.8). The mixtures were mixed well and heated up to 100 °C for 10 min in a water bath in order to obtain a light green color solution. The solutions were cooled to room temperature and the absorbance was measured with a UV-vis spectrophotometer from 600 to 800 nm. The concentration of as-made dialysis solution was determined to derive a calibration curve.

Henrietta Lacks (HeLa, human cervical cancer) cells were cultured in DMEM (containing 10% FBS) in a humidified 5% CO_2_ atmosphere at 37 °C. The in vitro cytotoxicities of PDA-PEG and PDA-PEG-CP on cells were tested as follows. Cells were seeded at a density of 8000 per well on a 96-well plate overnight and incubated in 100 μL of medium containing nanoparticles for 24 h. The cells were rinsed with PBS twice before adding fresh medium. An 808 nm diode laser at a power of 2 W was used to irradiate cell plates for a certain time to produce photo-thermal effect. The temperature of the solution was measured with an infrared camera. After irradiation, cells were incubated for another 24 h; MTT assay was used to examine cell viability as follows. An amount of 10 μL of MTT (5 mg/mL in PBS) was added and cells were incubated for 4 h in the dark. The medium was then replaced with 100 mL of DMSO and the absorbance was monitored using a microplate reader at 550 nm. The cells treated with the same procedure without adding MTT were used as the background control. The cell viability experiment was repeated three times and four parallel wells were used for each group.

## 3. Results and Discussion

**Figure 1:**
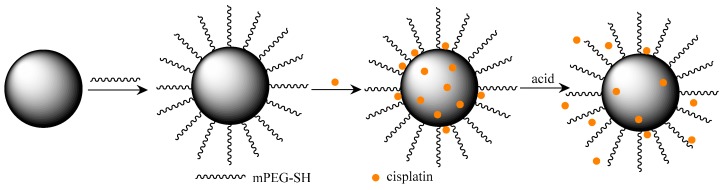
Scheme of loading cisplatin onto polyethylene glycol modified polydopamine nanoparticles (PDA-PEG-CP).

**Figure 2:**
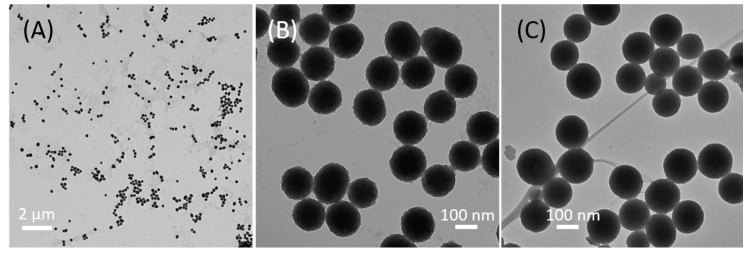
TEM images of polydopamine nanoparticles (PDA NPs) (**A**,**B**) with different magnifications and PDA NPs loaded cisplatin (PDA-PEG-CP) (**C**).

Figure 1 shows the procedure of making PDA-PEG-CP nanoconstructs. PDA-NPs were made by oxidation and polymerization of dopamine in an alkaline solution, where the color of the solution rapidly turned to yellow (oxidation) and gradually changed to dark (polymerization). Figure 2a,b show the TEM image of PDA-NPs, where the nanoparticles with uniform size (148 nm) are obtained. The nanoparticles of this size remain in circulation for a long period of time, and efficiently accumulate in tumor tissues via enhanced permeability and retention (EPR) [32,33,34].

**Figure 3:**
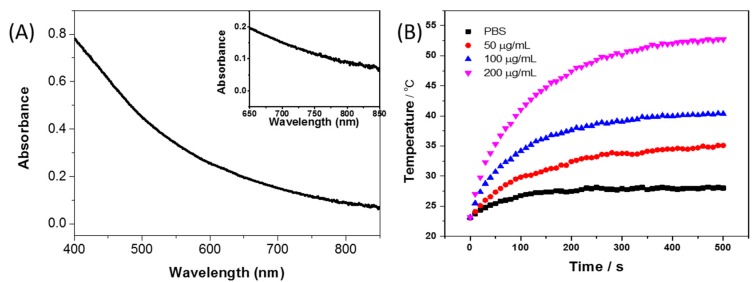
Absorbance spectrum (**A**) and enlarged image (inset) of PDA NPs. Temperature changes (**B**) of phosphate buffered saline (PBS) and PDA NPs of various concentrations under 808 nm laser irradiation (2 W/cm^2^).

Figure 3a shows vis-NIR spectra of PDA-NPs, where a strong absorption in the near infrared wavelength region can be found at 800 nm (inset). The photothermal effect of PDA NPs of various concentrations under 808 nm laser radiation is tested with PBS as a negative control. Figure 3b shows the temperature change profile of 0.2 mL PBS containing different concentrations of PDA-NPs, revealing the concentration-dependent photothermal effect. The temperature increased with the irradiation time for all the samples, and a higher concentration of PDA NPs leads to a more rapidly increasing temperature (Figure 3b). Take 200 μg/mL of PDA NPs for example; after 500 s of irradiation, the temperature increased by 29.6 °C, while the negative control (PBS) increased only 4.6 °C under the same condition. The rapid temperature change is due to the relatively high absorption coefficient (7.3 × 10^8^ M^−1^ cm^−1^) and photothermal efficiency (about 40%) at 808 nm of PDA NPs [26].

PDA-NPs were modified with mPEG-SH to enhance their circulation in physiological condition and facilitate their accumulation at tumor sites [35,36,37]. A Michael addition reaction was used to conjugate the thiol of PEG and α,β-unsaturated carbonyl on PDA [38]. The zeta potential of PDA decreased from −35.4 to −6.9 mV after PEGylation, confirming the successful conjugation of PEG on the PDA-NPs.

With abundant PDA groups on the surface, PDA-NPs can deliver the drug through electrostatic interaction, coordination and π–π interactions [39]. Cisplatin, a widely used anticancer drug was loaded onto the PEG-PDA NPs via the interaction between the platinum atom of cisplatin and catechol groups of PDA, and the nanoparticles were denoted as PEG-PDA-CP. The morphology and size of the NPs did not show obvious change between PDA and PDA-PEG-CP NPs, as seen in Figure 2b,c. The amount of cisplatin in the PEG-PDA NPs was measured to be 20% (*m*/*m*) using the OPDA method [31].

**Figure 4:**
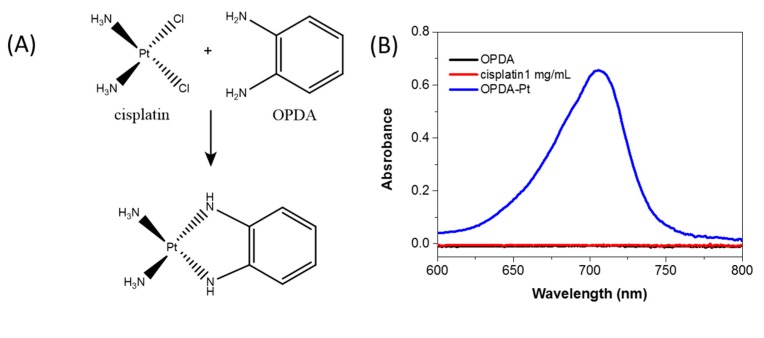

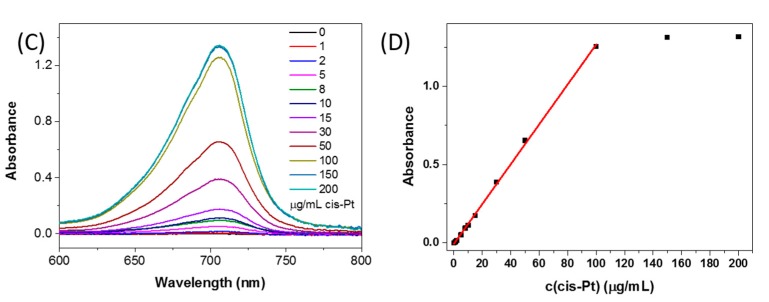
Reaction between o-phenylenediamine (OPDA) and cisplatin (**A**); the vis-NIR spectra of cisplatin, OPDA and the mixture of cisplatin and OPDA after incubation at 100 °C for 10 min (**B**); the vis-NIR spectra of the complexes at different concentrations of cisplatin (**C**); and the calibration curve used to determine the original concentration of cisplatin stock solutions from optical absorbance (**D**).

To study the release profile of cisplatin, PDA-PEG-CP solutions were suspended in PBS of different pH at 37 °C. The released cisplatin was quantified with an established method [31]. The formation of the complex of o-phenylenediamine (OPDA) and cisplatin was achieved by incubating OPDA and cisplatin in DMF spiked phosphate buffer at 100 °C for 10 min. The resulting solution showed a maximum absorbance at 705 nm, while neither OPDA nor cisplatin alone showed absorbance at 705 nm after the same treatment (Figure 4b). With the increasing concentration of cisplatin, the absorbance peak of the complex raised accordingly (Figure 4c), which is consistent with a previous report [31]. As a result, there is a good linear relationship between the absorbance intensity and concentration of 200 μL original cisplatin in the range of 0–100 μg/mL (Figure 4d).

**Figure 5:**
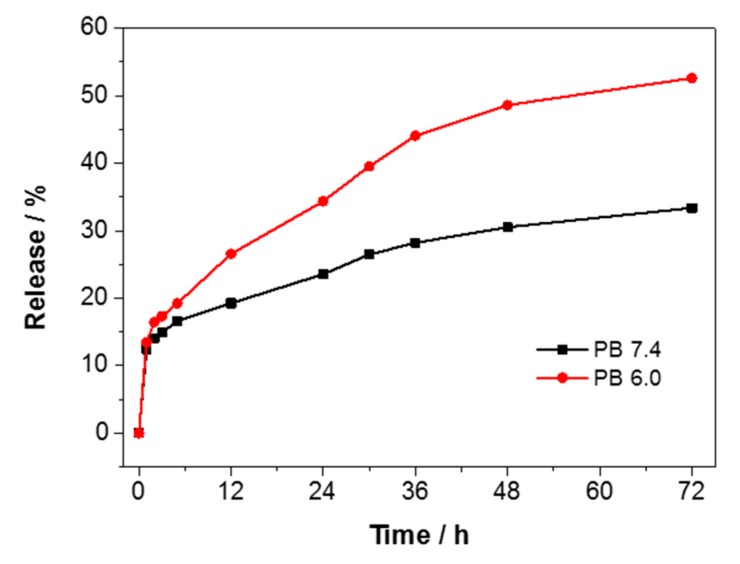
Cisplatin release curves against 10 mM phosphate buffer (PB) at different pH values (7.4 and 6.0).

It is essential to trigger cisplatin release from the drug delivery vehicles at tumor sites, thus keeping the overall therapeutic efficacy of the packaged drugs [40]. To this end, the release profile of cisplatin from the same batch of PDA-PEG-CP was evaluated through dialysis against saline buffered solutions at acidic (pH 6.0) and physiological (pH 7.4) conditions at 37 °C to mimic the acidic environment of the tumor site and cellular endosomes [41]. Cisplatin showed burst release within the first 1 h at both pH values. As expected, it showed a faster release at pH 6.0 than at 7.4 throughout the remaining period (Figure 5). After 72 h, 33.3% of the loaded cisplatin was released at pH 7.4; in comparison, at pH 6.0, about 45% cisplatin was released in the first 36 h and 55% release was obtained after 72 h. This acid-triggered drug release is due to the protonation of oxygen groups. In an acidic environment, H^+^ would attack the lone electron pair of oxygen, leading to decomposing of drug–PDA complexes [21]. More importantly, at pH 6.0, it showed sustained release during the 72 h and a continuous release can be observed from 48 to 72 h. The acid-enhanced drug release would enhance cancer therapy due to the acidic environment of most tumor cells [42,43].

**Figure 6:**
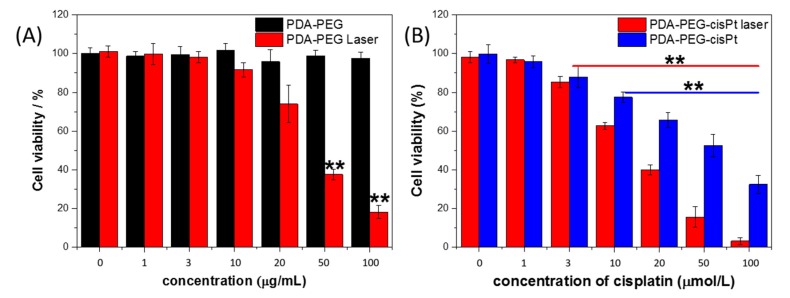
Viability of HeLa cells incubated at various concentrations of PDA-PEG (**A**) and PDA-PEG/CP (**B**) with or without laser irradiation (2 W/cm^2^). ** *p* < 0.01.

HeLa cells were used to assess the cytotoxicity of the drug delivery system. No obvious toxicity was observed after cells were incubated with PDA-PEG up to 100 μg/mL for 24 h (black histogram in Figure 6a). However, after 808 nm laser irradiation, apparent cell apoptosis can be obtained especially when the PDA-PEG concentration exceeds 20 μg/mL (red histogram in Figure 6a). Up to 85% of cells were destroyed when incubated with 100 μg/mL of PDA-PEG under 808 nm laser irradiation. It indicates great potential in PDA-based photothermal therapy. To examine the chemotherapy effect, PDA-PEG/CP at different concentrations of drug were incubated with HeLa cells for 24 h, then the cells were washed with PBS twice to remove non-internalized nanoparticles followed by 808 nm laser irradiation for 10 min. It was found that cell viability decreased with increasing PAD-PEG/CP concentration, and over 70% of cells were killed when incubated with 100 μg/mL of cisplatin loaded onto polydopamine nanoparticles (PDA NPs) (Figure 6b). As expected, more cells were destroyed with laser irradiation after incubation with PDA-PEG-CP. Cell viability of combined therapy was found to be lower than the summation of photothermal and chemotherapy alone. The remarkably improved therapeutic effect may be attributed to the photothermal effect which not only kills cancer cells, but also effectively enhances the delivery and release of the drug into cells for improved chemotherapy. The results indicate a synergetic effect when chemotherapy and photothermal treatment are combined.

## 4. Conclusions

A new nanoplatform based on nature-inspired polydopamine nanoparticles (PDA NPs) was created for combined photothermal and chemotherapy in cancer treatment. PDA showed great biocompatibility and molecular loading property, and enhanced photothermal conversion efficiency. A controlled amount of cisplatin was loaded onto PDA-PEG nanoparticles by chelation between platinum and catechol groups on PDA in minutes. This PDA-PEG/CP shows pH-dependent drug release, excellent biocompatibility and a remarkable synergistic effect. The results suggest that the PDA-based nanoplatform shows great promise for clinical application.
